# Patient Participation in Acute Surgical Wound Care: A Descriptive Qualitative Study

**DOI:** 10.1111/jan.16959

**Published:** 2025-04-28

**Authors:** Kita Liosatos, Georgia Tobiano, Brigid M. Gillespie

**Affiliations:** ^1^ School of Nursing and Midwifery Griffith Health, Gold Coast Campus Gold Coast Queensland Australia; ^2^ NHMRC Centre of Research Excellence in Wiser Wound Care, School of Nursing and Midwifery Griffith University, Gold Coast Campus Gold Coast Queensland Australia; ^3^ Gold Coast Hospital and Health Service Gold Coast Queensland Australia

**Keywords:** acute care, communication, nurse–patient relationships, patient participation, patient perspectives, post‐operative care, qualitative approaches, self‐care, surgical nursing, wound care

## Abstract

**Aim:**

To explore patients' experiences of participation in surgical wound care and provide an in‐depth understanding of their experiences with post‐operative wound care during and post‐hospitalisation.

**Design:**

A descriptive qualitative study.

**Methods:**

Adult participants who had undergone surgery within 30 days were purposively selected from two surgical wards at Gold Coast University Hospital. Seventeen semi‐structured phone interviews were conducted using a specifically developed and piloted interview guide. Textual data were analysed using inductive content analysis.

**Results:**

Three main categories were identified. The first category, ‘I didn't expect how distressing post‐operative wound care would be; it's tougher than I thought,’ highlights the significant and unexpected physical and emotional challenges participants faced, which initially hindered their engagement. The second category highlights the impact of healthcare professional interactions on patient participation, ‘I want to be involved, but conflicting advice and dismissive behaviour discourage me.’ The third category, ‘With my family's help, wound care got easier as I tried, learned, and recovered,’ illustrates how family support facilitated participants' independence and engagement over time.

**Conclusions:**

The spectrum of patient participation in surgical wound care is dynamic and impacted by environmental, physical and psychological factors. This research deepens understanding of patient participation by highlighting the importance of family support and a temporal perspective in patients' wound care journeys.

**Impact:**

Findings showed participants were unprepared for surgical wound care, greatly influenced by healthcare provider communication and family support, and evolved in participation as time passed and their wounds healed. Additionally, participants valued intent just as significantly as their behaviours and regarded even minor involvement as totally participative. These insights can inform strategies to improve patient participation in surgical settings.

**Reporting Method:**

SRQR (Standards for Reporting Qualitative Research).

**Patient and Public Contribution:**

No patient was involved in this study.

**Summary**

- Patient participation evolves, and as the wound heals, the patient overcomes challenges and gains experience.
- Patients value their intentions as much as their actions, and even minor involvement is considered fully participative.
- Patient participation exists on a spectrum of behaviours, with participants viewing even the smallest actions or intentions as meaningful forms of active involvement.

## 1 Introduction

Post‐operative wound complications, such as surgical site infections and surgical wound dehiscence, are a global concern for healthcare systems (Royle et al. 2023; Totty et al. 2021). With 11% of general surgical patients developing an infection within 30 days of surgery (Gillespie et al. 2021), surgical site infections in particular are among the most prevalent healthcare‐acquired infections, resulting in lengthier hospital stays and increased medical expenses (Andersson et al. 2010; McCaughan et al. 2018). These complications also have a significant impact on Australia's healthcare infrastructure and budget, costing the Australian healthcare system an estimated $323.5 million per year (Royle et al. 2023).

Yet, the consequences of surgical site infections and wound dehiscence extend beyond financial costs. Research indicates they lead to longer recovery times, increased pain and a higher risk of chronic conditions (Gibson et al. 2014; Lee et al. 2006; Limón et al. 2014). For older adults and those with comorbidities, these consequences can be particularly severe, with one study indicating that surgical site infections caused nearly one‐third of patient deaths (Astagneau et al. 2009). Beyond the physical toll, patients also face emotional distress, disruptions to daily life and loss of independence (Tanner et al. 2012; Walker et al. 2023). These complications also place financial burdens on patients and their families due to frequent dressing changes and limited access to subsidised care (Australian Medical Association 2022). In some cases, patients may turn to unproven alternative treatments due to barriers to affordability or access (Norman et al. 2015; Smith and McGuiness 2010), exacerbating their condition and creating further strain on healthcare systems.

## 2 Background

Reducing these risks requires effective post‐operative wound care, which calls for comprehensive wound management practices such as regular assessments, infection prevention and patient education on self‐care (Sinha 2019). In the hospital, clinical nurses primarily manage these practices (Kielo et al. 2019). However, upon discharge, the responsibility shifts to patients or their caregivers, who must follow treatment plans and attend follow‐up appointments (Garber et al. 2020; Lindsay et al. 2017). For patients with a surgical stoma, this transition can be particularly complex, as stoma care involves not only managing the stoma itself but also preventing complications such as peristomal skin breakdowns and surgical site infections. While wound care is a component of stoma care, the latter requires additional education and long‐term adaptation. Despite these complexities, traditional wound care approaches often undervalue patients' abilities to contribute to their recovery. Patients offer unique preferences and insights that, if implemented in care procedures, may dramatically improve their recovery. This shift requires embracing patient‐centred care, an approach that values patient input and autonomy in healthcare decision‐making (Kitson et al. 2013).

Patient‐centred care places patients' values and beliefs at the core of treatment decisions, fostering collaboration between healthcare professionals and patients (Kitson et al. 2013). Open communication and shared decision‐making allow treatments to be tailored to each patient's individual needs (Davis et al. 2005; Kwame and Petrucka 2021). A core component of this approach is patient participation, which involves actively engaging patients in their care decisions and self‐management. When patients are actively involved in their wound care, they are more likely to adhere to prescribed care routines and detect potential signs of complications earlier, reducing the risk of infections and delayed healing (Callender et al. 2021; Duhn et al. 2020).

While there is limited research on patient participation specifically in surgical wound care, evidence from other areas, such as chronic wound care and diabetic ulcer treatment, shows that patient participation can improve treatment outcomes, reduce complications and enhance overall quality of life (Kang et al. 2019; Tobiano, Marshall, et al. 2015; Ubbink et al. 2015). Opportunities for participation begin in the hospital, where patients can help determine dressing preferences, care routines and exposure to moisture. Research shows that patients who engage in these hospital activities are more likely to maintain similar self‐care routines after discharge (Tobiano et al. 2023). These self‐care routines may involve more physical components of wound care, such as wound cleaning, infection management, bandage change or removal and wound assessments (Sinha 2019; Ubbink et al. 2015).

## 3 The Study

### 3.1 Aim

This study aimed to explore patients' experiences of the spectrum of patient participation in surgical wound care and provide an in‐depth understanding of their experiences with wound care in an acute hospital and post‐discharge setting. The study questions included, ‘How do patients experience and participate in their acute post‐operative surgical wound care?’ and ‘What do patients perceive as barriers and facilitators to their involvement in their wound recovery?’. By exploring patient perspectives, we aimed to identify strategies that can enhance patient‐professional communication, improve self‐care training and ultimately reduce wound‐related complications.

## 4 Methods

### 4.1 Design

A descriptive qualitative study was conducted. This study was prepared following the Standards for Reporting Qualitative Research (SRQR) (O'Brien et al. 2014).

### 4.2 Theoretical Framework

This study adopted a constructivist interpretivist paradigm to explore patients' lived experiences of post‐operative wound care. It acknowledges that individual realities are shaped by personal beliefs, cultural background and healthcare interactions (Berger and Luckmann 2023). This approach allows researchers to investigate the complexities of wound care, including its psychosocial elements, and capture the breadth of patients' experienced realities of a little‐understood phenomenon (Rendle et al. 2019). According to Renjith et al. (2021), researchers can gain a deeper understanding of patient perspectives and design customised wound care strategies by utilising qualitative approaches such as interviews and observations, which can provide significant insights. To address the research aim and questions, a semi‐structured interview guide was specifically developed and pilot‐tested to understand how patients experience participating in their post‐operative surgical wound care.

### 4.3 Study Setting and Recruitment

This study was conducted at Gold Coast University Hospital (GCUH), a Magnet‐accredited facility with 750 beds that serves a population of over 665,000 people (Gold Coast Hospital and Health Service 2023). The Gold Coast University Hospital provides a wide range of surgical services, including general, orthopaedic, vascular and cardiothoracic procedures.

The study took place in two 28‐bed general surgical wards, catering to pre‐ and post‐operative patients with conditions such as colorectal, hepatobiliary and upper gastrointestinal disorders. Many patients in these wards were undergoing general surgery with a projected stay of more than 24 h. These wards were selected due to their high patient turnover and diverse caseload.

The study employed purposive sampling with maximum variation to select participants who would provide diverse perspectives on post‐operative wound care. Patients were chosen based on age, gender and type of surgery to ensure a broad representation of experiences. Recruitment and data collection occurred over two phases from July to August 2023. Based on prior qualitative nursing research conducted in comparable contexts, data sufficiency was estimated to occur at about 20 completed interviews (Kang et al. 2019; Latimer et al. 2013; Tobiano, Bucknall, et al. 2015). Recruiting over this threshold thus provided a safeguard against a predicted 20% attrition rate.

Recruitment was conducted systematically by reviewing ward handover sheets and coordinating with the Nurse Unit Manager to assess patient eligibility and well‐being. The first named author subsequently approached eligible participants during visiting hours. Informed consent was obtained through signed Patient Information Consent Forms, and a recruitment record was maintained to avoid duplicate approaches.

### 4.4 Inclusion and/or Exclusion Criteria

Table 1 shows the inclusion and exclusion criteria.

**TABLE 1:** Criteria for patient recruitment.

Inclusion criteria	Exclusion criteria
Surgical patients ≥ 16 years as per the legal age of consent for surgery.	Refusal/inability to give consent.
Have had an elective or emergency general, open or laparoscopic surgical procedure with a resultant surgical wound within the current period of hospitalisation.	Paediatric/neonatal, palliative, bariatric and pregnant/obstetric patients.
In‐patient in participating wards at the time of recruitment.	Patients discharged to residential aged care, respite or other care facilities.
Able to understand and speak English with or without an interpreter.	Patients receiving professional support services or hired assistance at home.
Physiologically well (as deemed by a healthcare professional) with full capacity to care for their own wounds.	
Capacity to give informed consent to research participation.	

### 4.5 Ethical Considerations

Ethics approval for the study was granted in June 2023 by the Gold Coast Hospital and Health Service Human Research Ethics Committee (HREC) (HREC reference number: HREC/2023/QGC/94783). The risk of harm was assessed as low. Informed consent was obtained from all participants after thorough verbal and written explanations of the study's purpose, procedures, risks and benefits. Participants could ask questions, consider their involvement and withdraw at any time without repercussions. Written informed consent was obtained before the interviews, along with recorded verbal consent for audio recording.

### 4.6 Data Collection

Data collection took place from 21 July 2023 to 23 August 2023. Participants were contacted 14 days post‐hospital discharge to schedule semi‐structured individual telephone interviews. Interviews were conducted approximately 17 days after discharge. In cases of unavailability, participants received two follow‐up phone calls and voicemail messages. After three attempts without response or at the end of the collection period, participants were withdrawn from the study. Demographic details, contact information and preferred interview times were gathered during recruitment.

A semi‐structured interview guide was developed to gather detailed responses about patient participation in wound care. This interview guide was informed by previous research in wound care and patient participation and was developed under expert supervision (Eldh et al. 2010; Thorup et al. 2018; Tobiano et al. 2023; Walker et al. 2023).

To ensure that the questions were clear, relevant and understandable, the main author held informal discussions with laypeople who had prior surgical experiences. The initial draft underwent pilot testing with eight individuals from various health literacy levels, and then refined based on personal reflections, feedback and expert input. This process ensured the final interview guide was practical and aligned with the study's research aims. Participants were asked a series of pre‐approved questions about their wound care experience, including how they participated in their care, what helped or hindered their involvement, their relationship with healthcare professionals and how well they felt informed about the process. Prompts like ‘Could you give me an example?’ and ‘Can you tell me more?’ were added to encourage participants to elaborate on their experiences. The guide was further pilot tested with three conveniently sampled participants who met the inclusion criteria but would be excluded by the time recruitment occurred. No further modifications were made before ethics approval. The full semi‐structured interview guide appears in Appendix A: Table A1.

Contact summary forms were used to document notes during and after each interview session. Interviews were audiotaped and transcribed verbatim using Microsoft 365's automated transcription service. This solution was chosen because it is practical, efficient, cost‐effective and complies with data protection laws such as the Health Insurance Portability and Accountability Act of 1996 (HIPAA), by using strong encryption algorithms to safeguard data during transmission and storage. To protect privacy and confidentiality, participants were assigned unique identification numbers, with all identifiable information stored separately from de‐identified data in locked or password‐protected locations.

### 4.7 Data Analysis

The data analysis was conducted using an inductive content analysis method, based on Elo and Kyngäs (2008), to capture both individual and collective participant experiences. The primary analysis was carried out by the main author, who met with the two other authors regularly to shape and refine the codes and categories during this stage.

Elo and Kyngäs (2008) identify preparation and organisation as the initial steps of content analysis. In the preparation phase, the main author immersed themselves in the data through transcript verification and revision. Interview recordings were transcribed verbatim using Microsoft 365 and compared against the original recordings to verify accuracy. Transcript revision was performed as necessary to maintain fidelity to the participants' words and meanings. After confirming the transcripts accurately reflected the recordings, we proceeded to the second stage of inductive content analysis.

The second stage involved open coding, which attaches descriptive codes to relevant text units to characterise the text's substance. The units of text could consist of single words, phrases, sentences or whole paragraphs relevant to the study's aim. The program NVivo 12 (QSR International) was used to apply codes to text, manage the textual data and display coded units for grouping.

The final step, abstraction of data, involved grouping comparable codes into subcategories, which reduced the overall number while preserving the coherence and authenticity of participants' voices. This entailed a systematic approach, particularly as several iterations were needed before we were satisfied the original codes' essence was preserved. These repeated attempts helped the main author deepen their immersion in the data and identify connections that were previously missed. Treating these groups as codes themselves facilitated the identification of patterns and recurrent themes during their examination. Further refinement and classification required grouping and refining subcategories to reveal subtleties in the data, which were then developed into comprehensive and relevant themes that served as basic categories. These findings served as the foundation for developing key themes on patient participation in post‐operative wound care.

### 4.8 Rigour and Reflexivity

Rigour in this study was achieved through adherence to credibility, dependability, transferability and confirmability (Lincoln and Guba 1985), along with researcher reflexivity. These criteria were implemented to ensure that the findings accurately represented the experiences and perspectives of participants. To maintain credibility, regular team meetings were conducted throughout the data analysis process to discuss emerging themes, compare findings and validate interpretations. This ongoing dialogue among the research team helped ensure that researcher bias was minimised and that the participants' original voices remained clear and authentic (Forero et al. 2018). Dependability meant having at least two or more researchers involved in the research design, data collection and analysis to ensure accuracy and consistency in the findings. Version control systems were established for all exchanged documents, meeting minutes and workday diaries, forming an audit trail to provide transparency and reliability. Transferability was enacted by providing detailed descriptions of the study's context, participants and methods, allowing readers to assess whether the findings might apply to their own clinical environments. Furthermore, all data analysis tables of categories and subcategories were included in the results, enabling readers to assess the results' suitability for their clinical settings. Confirmability of the data was ensured by enacting safeguards such as a specifically designed and tested interview guide, criterion‐based purposive sampling and gatekeeper assessments of participant eligibility. Finally, researcher reflexivity involved the author's ongoing reflection on personal biases and neurodivergent perspectives, and their potential impact on communication and data interpretation. They engaged in documented self‐reflection through journalling, maintained immersion in the data and sought feedback from both supervisors and laypeople.

## 5 Findings

### 5.1 Characteristics of Participants

Of the 30 participants who met the inclusion criteria, 26 consented and were recruited. Of these, 17 were interviewed, resulting in a 34.6% attrition rate. Reasons for drop‐out included non‐response (*n* = 4), incorrect phone numbers (*n* = 2), withdrawal of consent (*n* = 2) and one becoming ineligible due to professional support.

As seen in Table 2, the sample consisted of mostly male participants (*n* = 9), with a mean age of 60 years (SD: 15.2, range 22–83). Most had experienced surgery before (*n* = 12), and all required at least 1 day of hospitalisation. Thirteen participants underwent major surgeries, while the remainder had minor procedures. Five participants required a surgical stoma. Over one‐third underwent open (*n* = 8) or converted (*n* = 4) surgical procedures, resulting in larger wounds that required stitches along with keyhole wounds. Surgical details were derived from patient self‐report and clinical handover notes, which varied in specificity.

**TABLE 2:** Summary of participant characteristics (*n* = 17).

Characteristics	*n* (%)
Ward location	
Ward A	10 (58.8)
Ward B	7 (41.2)
Sex	
Male	9 (52.9)
Female	8 (47.1)
Age	
20–29	1 (5.9)
40–49	2 (11.8)
50–59	7 (41.2)
60–69	3 (17.6)
70–79	2 (11.8)
80–89	2 (11.8)
Highest level of education	
Lower secondary education (year 7 to year 10)	3 (17.6)
Upper secondary education (year 10 to year 12/13)	3 (17.6)
Post‐secondary non‐vocational education (TAFE)	4 (23.5)
Tertiary education (university, higher education)	7 (41.2)
Level of employment	
Part‐time work (15–34 h per week)	1 (5.9)
Full‐time work	6 (35.3)
On temporary leave (education leave, medical leave)	2 (11.8)
Retired/not working	8 (47.1)
First surgery	
Yes	5 (29.4)
No	12 (70.6)
Type of surgical approach	
Open	8 (47.1)
Laparoscopic	5 (29.4)
Both/converted to open	4 (23.5)
Surgical category	
Minor	4 (23.5)
Major	13 (76.5)
Surgery resulted in a stoma	
Yes	5 (29.4)
No	12 (70.6)

At the interview, 15 participants had healed wounds; two were recovering from infections. While 13 participants were able to independently care for their wounds, four participants still needed assistance from family or healthcare providers due to challenges like wound location, complexity and chronic health complications. Interviews lasted between 10 and 49 min (mean: 25 min, SD: 9.8).

### 5.2 Main Findings

**FIGURE 1:**
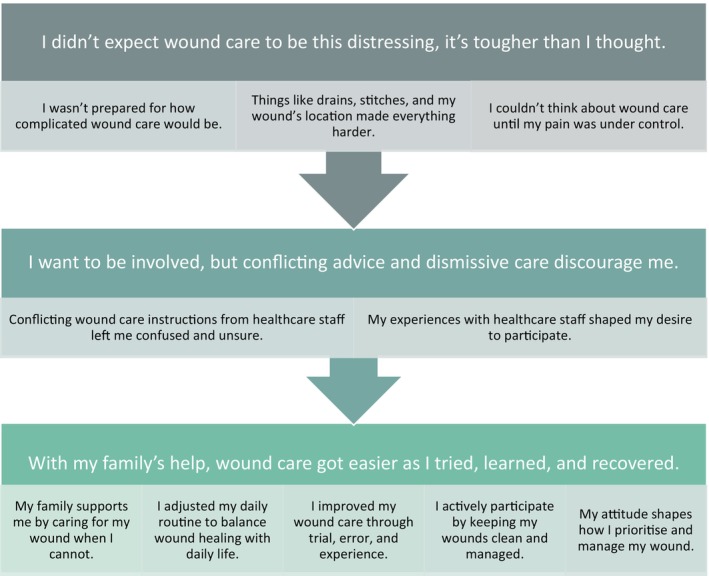
Categories and sub‐categories within each domain.

Figure 1 presents the three inductively identified categories and ten subcategories, providing a rich chronological timeline of patients' experiences of participating in post‐surgical wound care. Initially, patients encounter immediate barriers that must be addressed before engaging in wound self‐care. This is followed by a period of adaptation in which patients create relationships with healthcare providers and seek relevant information. Finally, patients reach the point when they actively manage their wounds, which becomes simpler with time and support. Findings are presented using direct quotes, with patients' reported gender and age range for demographic context.

#### 5.2.1 Theme 1: I Didn't Expect Wound Care to Be This Distressing; It's Tougher Than I Thought

In the first category, participants faced unexpected physical and emotional challenges alongside their surgical wounds. At this stage, many participants had not yet adjusted to the wound or developed coping strategies to manage the complexities of participating in post‐operative wound care. Challenges, such as the intensity of the experience, physical discomfort and disruption to regular routines, all contributed to an atmosphere of post‐operative shock and confrontation.

##### 5.2.1.1 I Wasn't Prepared for How Complicated Wound Care Would Be

This abrupt introduction to participants' recovery was often jarring, and ‘a bit of a shock’ (P11, M, 60s) upon waking from surgery. Many participants felt unprepared for the complexities of wound care, particularly when their expectations did not align with reality: ‘I thought originally we were going to do [wound care] at home, but unfortunately it was a little more complicated than what mom and I were prepared to do’ (P6, M, 20s). For some patients, wound care was a new and unpredictable challenge even when they had additional medical considerations, such as a stoma. While stoma care had its own unique requirements, participants still reported that managing their surgical wounds was a separate and often a more distressing experience: ‘I wasn't scared before the surgery, but I was in shock afterwards because of how painful the wound was.’ (P2, F, 50s). Unanticipated challenges both physically and emotionally affected participants' abilities to recover and eventually participate in their care, and led to recurrent feelings of frustration, anxiety and helplessness. However, not all revelations were negative. Some participants considered themselves as being ‘one of the lucky ones’ (P2, F, 50s; P17, M, 70s), as the wound itself ‘healed quickly’ (P2, F, 50s; P5, M, 50s) and ‘didn't itch’ (P11, M, 60s; P16, M, 50s), ‘which was a bit of a surprise’ (P16, M, 50s).

##### 5.2.1.2 Things Like Drains, Stitches and My Wound's Location Made Everything Harder

Participants found wound recovery challenging not only due to the complexity of the wounds themselves but also because of factors external to the wounds, such as the presence of drains, stitches, scarring and the location of the wound. They regarded these aspects as ‘complex’ (P6, M, 20s) and ‘challenging’ (P2, F, 50s), causing them to feel overwhelmed, cautious and unwilling to participate in wound care: ‘My concerns scared me away from dealing with the wound […] I could easily do more damage than good’ (P6, M, 20s). Some participants required assistance because they could not see their wounds or fainted at the sight of them. When asked about their wounds, many participants agreed: ‘The drain wound was the worst out of all of them.’ (P1, M, 50s).

##### 5.2.1.3 I Could Not Think About Wound Care Until My Pain Was Under Control

The biggest immediate confrontation participants encountered was wound pain, which dominated their lives and affected their well‐being. Participants reported ‘losing sleep’ (P11, M, 60s), ‘not caring’ (P1, M, 50s) about healthcare professionals' education, being ‘restless’ (P7, M, 50s) and ‘hardly doing anything’ (P14, M, 80s) because of their constant pain. Even the simplest tasks demanded caution: ‘You're conscious of absolutely everything you're doing because you pay for it’ (P11, M, 60s). Participants' apprehension about pain resulted in a broader trend where wound care participation was deemed ‘out of the question’ (P13, F, 60s) until the pain was adequately addressed. In addition to reducing participants' physical suffering, effective pain management also increased their willingness to seek healthcare advice: ‘I was very happy to listen and take any advice that I got that was gonna make it less painful for me’ (P11, M, 60s). While pain management helped participants engage with their recovery, it also presented its own challenges. Prioritising pain relief was a ‘main concern’ (P7, M, 50s) for participants, but post‐surgery anaesthesia left participants feeling disoriented and uncertain (P2; P11). Despite these complexities, timely pain relief before dressing changes was considered crucial as the pain ‘would have been too much to handle’ (P14, M, 80s). Although participants did not explicitly link pain relief to improved wound care participation, their experiences suggested that once pain was managed, they could gradually refocus on their recovery.

#### 5.2.2 Theme 2: I Want to Be Involved, but Conflicting Advice and Dismissive Care Discourage Me

The second category signified a pivotal stage in patients' journey, as they transitioned from the initial shock of the wound to actively navigating their roles in wound care, seeking information and engaging in collaboration with healthcare professionals. The influence of healthcare professionals on patients' participation in surgical wound care was noted through participants' discussion of complex relationships and information‐sharing practices in the hospital. Participants wanted collaboration and were actively empowered when healthcare professionals offered beneficial relationships and clear, consistent information. Conversely, participants felt frustration and uncertainty when encountering conflicting information or negative interactions, which impeded their motivation and ability to engage effectively in their care.

##### 5.2.2.1 Conflicting Wound Care Instructions From Healthcare Staff Left Me Confused and Unsure

Participants received inconsistent guidance and conflicting information from healthcare professionals. As a result, several individuals were confused, passive and noncompliant. While most participants could not recall receiving clear post‐surgery wound care instructions, those who did reported differences in teaching methods among healthcare providers, as well as divergent advice from surgeons and nurses: ‘Doctors' thoughts weren't written down, so the nurses would go by their own experience.’ (P2, F, 50s). However, some participants found nurses' information practical and relevant, extending beyond conventional wound care:

They explained to me about hernias, and to prevent them while coughing you need to grab the pillow. They even had them taped up there to be ready, and you're holding them into your stomach so it [the wound] doesn't rupture. It makes a big difference. (P11, M, 60s)

##### 5.2.2.2 My Experiences With Healthcare Staff Shaped My Desire to Participate

Participants' experiences with healthcare professionals varied, with negative interactions instilling distrust, anxiety, reluctance and passivity among some. For instance, participants felt brushed off, shamed and minimised by rude and authoritarian staff: ‘[the surgeon's] bedside manners were absolutely terrible. She just came to rip the dressing straight off without any care whatsoever’ (P8, M, 40s). These interactions hindered effective communication and eroded trust in professionals' judgements, impacting participants' confidence in managing their wounds. Some participants chose to defer the responsibility of wound care to staff, viewing their hospital stay as a time to be cared for by professionals: ‘I was in their [staff] care, so I just let them do it. I let them have it.’ (P15, F, 50s). Other participants felt passive due to a lack of knowledge and relied on nurses for wound care instructions to continue at home. However, they felt they had to balance their desire to learn wound care with nurses' demanding schedules, which could erode the relationship: ‘[the nurses] were adamant that they were doing it and that was that’ (P1, M, 50s).

In contrast, encouraging and cooperative interactions with medical professionals inspired participants to take an active role in their treatment and feel comfortable caring for their wounds at home. Participants complimented nurses for their attention and resourcefulness, crediting their recovery to their persistence: ‘They made me do it myself… I reckon without [the nurses] I probably wouldn't have been here, or I would have lost my leg’ (P5, M, 50s). Stoma care nurses were primarily responsible for teaching some participants how to manage their stomas, but participants' stories revealed that their role went beyond that. Several participants noted that these nurses also provided valuable guidance on surgical wound care, self‐care and long‐term management. As a result, participants with stomas felt more equipped to take an active role in their surgical wound recovery: ‘I believe they got me to a position that I knew what I needed to do’ (P11, M, 60s). Following discharge, this autonomy expanded beyond wound care to include proactive healthcare management, such as seeking medical attention:

I made an appointment this morning to see [the doctor] today. I went in and said ‘Look, there's a hole on my left side and it was bleeding on Thursday.' (P3, F, 70s)

#### 5.2.3 Theme 3: With My Family's Help, Wound Care Got Easier as I Tried, Learned and Recovered

The third category illustrates participants' growing independence and active participation in their wound care, indicating a shift towards proactive self‐care. At this point, they were discharged and expected to care for their wounds at home. Initially, they struggled with the complications of wound treatment and relied largely on medical professionals. However, with time, effort and family support, they increasingly found the process more manageable, which influenced the extent of their active participation. Participants articulated a variety of approaches to participating in their care, adjusting their daily routines, learning from their experiences and contextualising their wound care. These variations were intentional and followed by a justification, highlighting the significance of understanding why they were participating. For participants, the increasing ease of participation enabled them to continue to actively engage and heal.

##### 5.2.3.1 My Family Supports Me by Caring for My Wound When I Cannot

Participants constantly emphasised the importance of family support during their wound care journey, both physically and emotionally. Family members played an important role in wound treatment, domestic activities, nutrition and transportation. In other cases, family members provided direct treatment to participants' wounds and advocated for them when complications arose: ‘She loves me, and she wants to look after me because she wants to, not because […] she has to’ (P17, M, 70s). Beyond physical involvement, participants recounted the impact of emotional support from ex‐partners, neighbours and even children:

My 11‐year‐old boy, you know, would lay with me in bed and he'd rub my tummy where the scar was […] It was just a nice sentiment and yeah… I guess nicer connection between him and I. (P8, M, 40s)

##### 5.2.3.2 I Adjusted My Daily Routine to Balance Wound Healing With Daily Life

Participants described proactively adapting their daily routines to align with personal wound‐healing goals. They recognised discomfort, sleep disturbance and weakness caused by their wounds limited them from daily activities and worked to establish a sense of normalcy in their routine. Some enlisted the help of family members to perform chores, while others changed their clothing or surroundings to suit them: ‘I made sure my clothes were folded down. So, I didn't have any elastic on the wounds or anything’ (P10, F, 80s). Participants' approaches to wound care during showers varied; some only utilised showers to inspect their wounds, others cleaned their wounds with soaps or antibiotic solutions, some treated their wounds indifferently, while others avoided showers in fear of getting their bandages wet.

##### 5.2.3.3 I Improved My Wound Care Through Trial, Error and Experience

Participants refined their wound care methods through trial and error, gradually improving their independent care practices: ‘I've learned something every day about my wound care’ (P1, M, 50s). Participants were willing to learn from their mistakes and adapt their approaches as they gained experience and familiarity with their wounds: ‘Well, nobody's born with all the knowledge… you gotta go out and either experience or ask about it’ (P1, M, 50s). For many participants, prior experiences of surgery and infection helped contextualise their healing and prepare them for complications: ‘Being a mother with three kids, you do learn what's infected and what's not’ (P10, F, 80s).

##### 5.2.3.4 I Actively Participate by Keeping My Wounds Clean and Managed

Participants demonstrated their self‐efficacy by describing how they monitored their wounds, personalised bandaging approaches and used alternate wound protection and management measures. While some chose hands‐on measures such as changing bandages with saline and gauze, others took a more relaxed approach: ‘[I] just kept them clean, kept them aired’ (P7, M, 50s). Although participants had varying practices and preferences for wound care, they were not just following orders to participate; every act they described was intentional and followed by a justification. It mattered significantly for participants to understand why they were participating:

I looked after [the wounds] quite well when I came home knowing that if it didn't, I could be back in, or get an infection and it'll put me back on my behind again. (P1, M, 50s)

##### 5.2.3.5 My Attitude Shapes How I Prioritise and Manage My Wound

Many participants expressed a strong sense of personal responsibility when describing their wound care. They recognised the risks of ignoring their wounds and addressed wound care with a resilient attitude: ‘It's your body. No one's really gonna look after it except for yourself, and you don't want it to get worse than what it already is’ (P15, F, 50s). However, their prioritisation and approach to wound care differed between individuals. The broader context of their health management often shaped the extent of their active involvement in the process:

With the whole thing of the operation and everything and looking down the barrel of starting the chemo and also being one day a week of intravenous, I started to think, Jesus, I'm not gonna be able to handle it. You know, like. I started to worry about it. (P11, M, 60s)

Participants with more complex or infected wounds prioritised their recovery highly, while participants with simpler wounds reflected feeling ‘blasé’ regarding their lack of participation. Despite this, they maintained a daily check on their wound and remained unworried when it appeared fine: ‘If it ain't broke, don't fix it. So, if you check [the wound], it's not broken? Leave it alone’ (P16, M, 50s). Despite wound care being a lower priority within participants' broader healthcare management, they maintained a proactive mindset by assessing their wounds daily.

## 6 Discussion

Our study contributes to the literature by advancing the understanding of patient participation in post‐operative wound care. More specifically, our study builds upon our integrative review's findings (Liosatos et al. 2024) by offering detailed insights into the challenges patients face and how their engagement evolves over time. Although participants faced a variety of post‐operative difficulties, our analysis excluded challenges unrelated to wound healing, like stoma care, gastrointestinal changes or nutritional concerns, and focused specifically on their surgical wounds. Initially, participants struggled with the physical and psychological effects of their wounds, as their discomfort and emotional response needed to be addressed before they could engage in wound care. The transition to active self‐care was heavily influenced by the support they received from medical professionals. Some participants experienced setbacks due to inconsistent communication or inadequate preparation from their care team. Finally, participants reached the point of independence, with family support, the wound's healing and individual learning catalysing this process. Our knowledge of patients' involvement in post‐operative wound care has developed from a general classification of influencing elements to a comprehensive, context‐rich comprehension of the patient experience.

We found that participants' challenges were often compounded by the physical symptoms and emotional toll associated with surgical wounds. These wound‐related concerns, including pain, visibility and fear of infection, shaped their psychological readiness for self‐care. Participants were frequently unprepared for the realities of managing their wounds post‐operatively, which left them preoccupied with immediate pain and anxiety rather than long‐term wound care management. This finding reflects previous research highlighting that the burden of wound care is psychological as well as physical (Goh and Zhu 2018; McCaughan et al. 2018). Consistent with our integrative review, participants reported challenges related to visible wounds, physical discomfort and anxiety about complications (Liosatos et al. 2024). Accessible wounds are easier to care for (Goh and Zhu 2018), but their constant visibility can be distressing. McCaughan et al. (2018) found that visible surgical reminders, such as drains, stitches and scarring, heightened feelings of vulnerability and helplessness in participants. These factors often deterred participants' self‐care, particularly when combined with sensory disturbances like nausea and dizziness. Research shows that fear of pain, infection and wound deterioration can further discourage patient participation (McCaughan et al. 2018; Wei et al. 2024). Hence, prompt identification and treatment of these issues is critical, because if left unresolved, patients may refuse to participate in their care altogether.

Our findings align with earlier research on how poor pain management can lead to emotional distress, exhaustion and a slower recovery (Probst et al. 2023; Tanner et al. 2012). Suboptimal pain management can cause confusion, disorientation, increased complications and slower recovery (Barrington et al. 2014; Bettelli 2017). When combined with insufficient sleep and restricted activity, constant pain can lead to emotional distress, exhaustion and a perceived lack of control (Probst et al. 2023; Tanner et al. 2012). Conversely, pain relief medications may induce numbing or amnesiac effects, impairing alertness and self‐care (Liosatos et al. 2024). In wound care, effective pain management is challenging; Wei et al. (2024) found that nearly one‐third of chronic wound patients fear dressing changes due to pain, leading to negative expectations and increased mental distress (Probst et al. 2023). Given the difficulties of managing pain associated with open wounds, patients' concerns about pain management are warranted.

Our findings also suggest that patients' prior wound care experiences significantly affect their ability to cope with post‐operative challenges. Participants with prior surgical experiences often used their experiences as a reference point to assess their current recovery; previous research has found this practice more common among those with substantial healthcare backgrounds (Kelly et al. 2016). However, such experience is not enough to prepare patients for the specific needs following complex procedures, which leads to unmet expectations and challenges in recovery (Powell et al. 2016). We found that participants who underwent complex open surgeries were ill‐prepared for increased pain and psychological distress, regardless of their prior experience. This reveals a gap in preoperative education regarding post‐operative challenges, especially for first‐time patients or patients undergoing more complex surgical procedures.

Our findings affirm the critical role of patient‐professional communication in wound care, particularly in managing post‐surgical recovery. Our prior review established the negative effects of inconsistent patient‐provider interactions (Liosatos et al. 2024), but this study unexpectedly revealed siloed, non‐collaborative communication among healthcare professionals themselves. Participants reported, for example, that they saw doctors who unbandaged their wounds and advised them to air their wounds out, but ward nurses soon rebandaged the wounds to reduce the risk of infection. In addition to confusing the participants, this inconsistent strategy risked post‐operative complications and potentially wasted important healthcare resources. Doctors are traditionally viewed as medical authorities (Ennis 2012), while wound care is usually associated more with nursing, as it is perceived as unappealing and less prestigious (Galazka 2021). This perception may lead doctors to neglect wound care education (Welsh 2017). Gillespie et al. (2014) also found that nurses primarily rely on informal sources, such as coworker advice and personal experience, while evidence‐based education recommended in national wound care standards is not consistently pursued among healthcare professionals (Buus et al. 2021). These dynamics may have contributed to the study participants inadvertently receiving conflicting information.

Not all communication, however, was siloed; our findings identified the primary impact of effective collaboration through the unique influence of stoma care nurses on patient participation. Every participant who received a stoma was autonomous, capable and in control of their wound care, which they attributed to the education from stoma care nurses. In contrast, those receiving conflicting information from medical staff adopted a passive approach and postponed active self‐care until after discharge. The degree of evidence‐based training and resources available to health professionals may impact this discrepancy. Stoma care nurses are highly specialised experts in complex stoma and wound care (Bird et al. 2023). In addition to providing standard wound care, their duties also involve encouraging early patient involvement in addressing stoma concerns, providing long‐term psychological support and delivering comprehensive patient education (Steinhagen et al. 2017). On the other hand, general healthcare professionals like doctors and ward nurses typically lack this level of training in stoma or wound care (Dunne et al. 2021). The strategies used by stoma care nurses to promote patient autonomy and active wound care engagement may be the subject of future studies.

Our participants described the crucial role of family support in patient participation. For example, participants who had regular assistance from family members demonstrated greater confidence in proactive wound care. This aligns with a systematic review conducted across 10 countries; regardless of setting, culture or country, families consistently worked to provide a supportive environment (Whitehead et al. 2018). Although factors like wound location, visibility and pain influenced participation, accessibility in our study was not a concern; all participants immediately listed family members willing to assist. Our findings support the notion that family is essential for both emotional and practical aspects of care, especially for patients lacking the physical ability or confidence to manage their wound care independently (Zhu et al. 2023). The absence of this support becomes especially significant, as patients may find wound care much harder to manage on their own.

While most participants in our study were able to reach an autonomous role in wound care within 2 weeks, it is important to acknowledge that not all patients may be able to achieve full independence by this time. Previous research shows that the time required for patients to achieve independence varies (Wu et al. 2016). Issues like inadequate support networks, limited health literacy, difficult‐to‐access wounds, poor communication skills and underlying medical disorders could contribute to this delay (Ryan and Post 2022). Nonetheless, every participant in our study actively participated in aspects of their care that were within their capabilities. Moreover, participants with complex or inaccessible wounds were frequently overwhelmed; but they remained engaged in manageable aspects of their care, such as reaching out to family for support. This aligns with Yao et al. (2021), who found that patients' intentions to engage in surgical site infection prevention activities were directly impacted by elements such as self‐efficacy, participation attitude and social support. These behaviours imply that patient involvement in wound care is a spectrum of engagement where even minor actions can have a significant impact on recovery.

### 6.1 Limitations and Strengths of the Work

This study's main strength lies in its rigorous use of qualitative methodology with in‐depth semi‐structured interviews, which captured a detailed insight into how patients interact with their wound care. The richness of the data collected ensured data sufficiency, which enabled a comprehensive inductive content analysis. However, a limitation identified in the interviews is that participants self‐reported their wound care activities, restricting data collection to actions they considered significant enough to disclose. While a more structured interviewing method might have prompted participants to talk about overlooked behaviours, doing so could unintentionally sway responses and establish researcher bias. To mitigate this, the author practised reflexivity, intentionally setting aside their preconceived biases and assumptions. They also maintained a reflective notebook throughout data collection and analysis and engaged in frequent meetings with supervisors.

Despite our attempts to achieve a sample size with maximum variation, the study's sample was constrained by time, location and cultural context. As an unfunded study, the author was limited to locations and times that were logistically feasible, which limited the ability to recruit a more varied sample. Most participants were of similar cultural and socioeconomic backgrounds, predominantly English‐speaking and urban dwelling. Additionally, the impact of the 2020 global coronavirus pandemic on Australia's healthcare system further restricted the participant pool, with many elective surgeries still cancelled or postponed. Finally, while the study's location in a large city facilitated accessibility, it may have limited inclusion from more rural or remote areas. To address this limitation, we thoroughly described the study context, enabling readers to assess the transferability of the findings to other settings. Despite these constraints, the sample was diverse in terms of age, type of surgical wound, surgical experience and participants' perspectives.

### 6.2 Implications for Policy and Practice

The gaps in patient‐professional information sharing revealed by our research indicate a need for improved communication. Clinicians should consider implementing Enhanced Recovery After Surgery (ERAS) protocols, which prioritise pain management and comprehensive preoperative counselling (Jain et al. 2023). Preoperative counselling helps patients set realistic recovery goals, anticipate complications and actively participate in managing their care by preparing them for the reality of wound care (Buus et al. 2021). Simplified educational materials and the inclusion of family in care discussions can further aid comprehension and support throughout the recovery process (Oliveira et al. 2023; Walker et al. 2023). Educators should highlight wound management training in undergraduate nursing and medical programmes, with opportunities for multidisciplinary collaboration, such as including stoma care nurses, to strengthen clinician expertise.

### 6.3 Recommendations for Further Research

Finally, further research could explore how patients with more complex wounds, such as stomas or perineal wounds, or those associated with bariatric surgery, navigate wound care participation, particularly in relation to support needs and recovery expectations. Additionally, researchers should examine how cultural and socioeconomic factors affect participation in post‐operative wound care, particularly for under‐researched groups such as Aboriginal and Torres Strait Islander patients (Thompson et al. 2012). Comprehensive demographic surveys that capture cultural backgrounds and studies on socioeconomic status, education and healthcare access can provide a more complete picture. This approach can help develop culturally sensitive, patient‐centred strategies to improve participation and outcomes for diverse patient populations.

## 7 Conclusion

This study explored how patients participate in post‐operative wound care, identifying significant challenges they must overcome and the evolving nature of their involvement. Initially, patients faced psychological and physical barriers, but with support from family and healthcare professionals, their autonomy and self‐efficacy grew. Positive interactions with healthcare professionals, especially stoma care nurses and strong family support were instrumental in fostering participants' independence and active engagement in wound care. Exploring patient participation revealed a spectrum of behaviours and levels of engagement, all perceived as active involvement by the participants themselves.

## Author Contributions

**Kita Liosatos:** conceptualisation, methodology, formal analysis, investigation, data curation, writing – original draft, visualisation. **Georgia Tobiano:** conceptualisation, methodology, validation, investigation, resources, data curation, writing – review and editing, supervision, project administration. **Brigid M. Gillespie:** conceptualisation, methodology, validation, investigation, data curation, writing – review and editing, supervision, project administration.

## Disclosure

The authors have checked to ensure our submission conforms as applicable to the journal's statistical guidelines. The authors affirm that the methods used in the data analyses are appropriate for the study design and context, and all findings have been implemented and interpreted correctly. The authors take full responsibility for ensuring the accuracy and correct interpretation of the methods applied.

## Ethics Statement

Ethical approval was obtained from the Gold Coast Hospital and Health Service Human Research Ethics Committee. Informed consent was obtained from all participants prior to data collection.

## Conflicts of Interest

The authors declare no conflicts of interest.

## Supporting information


Data S1.

## Data Availability

The authors confirm that the data supporting the findings of this study are available within the article and its Supporting Information.

## Appendix A Semi‐Structured Interview Guide Development

**TABLE A1:** Semi‐structured interview guide.

Domain	Questions
Background/context	You have a surgical wound, what stands out the most about that experience?
Theoretical	2 Have you ever heard of ‘Patient Participation’, ‘Patient Empowerment’ or ‘Patient Involvement?’
Personal	What were some things you noticed about your wound after the surgery? How did you take part in your wound care after surgery? Prompt: please tell me some examples of ways you took part in your wound care in the hospital. Prompt: please tell me some examples of ways you took part in your wound care once home. Did the way you took part in your care, match your expectations? What helped you participate in your care? What hindered participation in your care? How much did the HCPs inform you of the wound care prior to surgery and what to expect afterwards? Did the information match up with your expectations? How would you describe your relationship with your HCPs? Did anything influence your wound care (both from HCP and their own self‐care). What is your overall view of your wound care experience these past 14 days?
